# Liquid Biopsy in Lung Cancer Screening: The Contribution of Metabolomics. Results of A Pilot Study

**DOI:** 10.3390/cancers11081069

**Published:** 2019-07-29

**Authors:** Sandeep Singhal, Christian Rolfo, Andrew W. Maksymiuk, Paramjit S. Tappia, Daniel S. Sitar, Alessandro Russo, Parveen S. Akhtar, Nazrina Khatun, Parveen Rahnuma, Ahmed Rashiduzzaman, Rashid Ahmed Bux, Guoyu Huang, Bram Ramjiawan

**Affiliations:** 1 Columbia University Medical Center, New York, NY 10032, USA; 2 Marlene and Stewart Greenebaum Comprehensive Cancer Centre, University of Maryland, Baltimore, MD 21201, USA; 3 Cancer Care Manitoba, Winnipeg, MB R3E 0V9, Canada; 4 Department of Internal Medicine, Rady Faculty of Health Sciences, University of Manitoba, Winnipeg, MB R3A 1R9, Canada; 5 Asper Clinical Research Institute & Office of Clinical Research, St. Boniface Hospital, Winnipeg, MB R2H 2A6, Canada; 6 Department of Pharmacology & Therapeutics, Rady Faculty of Health Sciences, University of Manitoba, Winnipeg, MB R3E 0T5, Canada; 7 Medical Oncology Unit A.O. Papardo & Department of Human Pathology, University of Messina, 98158 Messina, Italy; 8 Department of Medical Oncology, National Institute of Cancer Research & Hospital, Mohakhali, 1221 Dhaka, Bangladesh; 9 FastBios, House 12, Rd 14/C, Sector 4, Uttara, 1230 Dhaka, Bangladesh; 10 BioMark Diagnostics Inc., Richmond, BC V6X 2W8, Canada

**Keywords:** metabolomics, lung cancer, metabolomic fingerprint, NSCLC, polyamine, SSAT-1

## Abstract

*Background*: Lung cancer is the most common cause of cancer-related deaths worldwide. Early diagnosis is crucial to increase the curability chance of the patients. Low dose CT screening can reduce lung cancer mortality, but it is associated with several limitations. Metabolomics is a promising technique for cancer diagnosis due to its ability to provide chemical phenotyping data. The intent of our study was to explore metabolomic effects and profiles of lung cancer patients to determine if metabolic perturbations in the SSAT-1/polyamine pathway can distinguish between healthy participants and lung cancer patients as a diagnostic and treatment monitoring tool. *Patients and Methods*: Plasma samples were collected as part of the SSAT1 Amantadine Cancer Study. Liquid chromatography-tandem mass spectrometry (LC-MS/MS) was used to identify and quantify metabolite concentrations in lung cancer patient and control samples. Standard statistical analyses were performed to determine whether metabolite concentrations could differentiate between healthy subjects and lung cancer patients, as well as risk prediction modeling applied to determine whether metabolic profiles could provide an indication of cancer progression in later stage patients. *Results*: A panel consisting of 14 metabolites, which included 6 metabolites in the polyamine pathway, was identified that correctly discriminated lung cancer patients from controls with an area under the curve of 0.97 (95% CI: 0.875-1.0). **Conclusion:** When used in conjunction with the SSAT-1/polyamine pathway, these metabolites may provide the specificity required for diagnosing lung cancer from other cancer types and could be used as a diagnostic and treatment monitoring tool.

## 1. Introduction

Lung cancer is one of the most common types of cancer worldwide and is the most common cause of cancer-related death [1,2,3] in both men and women, accounting for 25% of cancer deaths. Based on the U.S. Preventive Services Task Force on Screening for Lung Cancer, approximately 90% of lung cancer patients die from the disease, in part because it is often not diagnosed until an advanced stage when the chance to receive curative-intent treatments is low. Radiographic screening using new technology with low exposure to radiations (low-dose computed tomography, LDCT scan) can reduce the risk of death from lung cancer and increase the possibility to be cured. Currently LCDT is routinely used for lung cancer screening in high-risk individuals.

Although the NELSON ( The Dutch-Belgian Randomized Lung Cancer Screening Trial (Dutch acronym: NELSON study ) trial has shown that LCDT screening has selectivity of 85% and a specificity of 99% in comparison with no screening [4], a recent study of the Providence VA Lung Cancer Screening Program showed that the overall false positive rate was 81% [5]. This very high rate required cumbersome follow up which included additional imaging or testing to confirm the results. There is a need for evidence-based biomarkers to support pre- and post-test risk assessment in order to optimize image-based screening further refining screening selection criteria to limit the costs and providing a post-test risk assessment capable of informing clinical decision making in the management of indeterminate pulmonary nodules [6]. Several protein-based, microRNA and DNA-methylation assays for lung cancer detection have been described in the literature or are under commercial development [7,8].

It is well understood that cancer cells have a fundamentally different metabolism than non-cancerous cells and this difference is manifested in the endogenous metabolites they produce [9]. Metabolomics is an emerging multi-disciplinary approach, which combines advanced analytical chemistry techniques with machine learning and statistical modeling to characterize and quantify thousands of metabolites found in tissues and biofluids. The use of metabolomic profiling has also been demonstrated in the differentiation of lung cancer stages as well as discrimination of chronic obstructive pulmonary disease (COPD) from lung cancer [9,10,11,12,13,14]. Cancer interception is the active way of detecting and treating cancer and carcinogenesis at earlier stages [15] and metabolomics has recently been applied to the discovery of tumor biomarkers for the diagnosis, treatment, and prevention of different solid tumors, including lung cancer [16]. The goal of metabolomics is to identify markers that can help distinguish between lung cancer and healthy patients, various lung cancer types and stages, and also aid in tumor detection [6,16]. Similarly, oncoproteomics evaluate protein modification in malignancy to identify biomarkers that can be used for detection, stratification or prognosis of cancer and cancer therapies [17].

Previous studies have reported the utility of the enzyme spermidine/spermine N^1^-acetyltransferase-1 (SSAT-1) [18] as a cancer detection tool [19,20,21]. SSAT-1 is a key protein involved in the synthesis and homeostasis of the polyamines spermine and spermidine [22]. These polyamines have specific roles in maintaining the membrane potential, controlling intracellular pH, and cell volume [23]. SSAT-1 is upregulated in breast, kidney, liver, lung and hematological cancers [19,23,24,25,26] as well as in glioblastoma [26,27,28,29]. Thus, further examination of metabolites within the polyamine pathway represents a potential tool for enhanced prediction in the diagnosis of cancer [21]. 

Amantadine is a US Food And Drug Administration (FDA)-approved drug for Influenza and Parkinson’s disease and is a specific substrate for SSAT-1 [30]. We have earlier reported the clinical utility of amantadine to detect elevated SSAT-1 activity by measuring increased concentrations of acetylamantadine in the urine of cancer patients [31,32]. Based on the upregulation of SSAT-1 in different types of cancer, we developed a customized assay to help explore whether (1) the concentrations of polyamine and other endogenous metabolites comprised of amino acids, biogenic amines, acylcarnitines and glycerophospholipids in serum of lung cancer patients could be used as a diagnostic test for lung cancer and (2) evaluate whether combining a panel of these new metabolites can further enhance and complement the SSAT1 amantadine assay performance for detection of lung cancers [32]. Here, we report a metabolic fingerprint determined by a customized liquid chromatography mass spectrometry (LC-MS) assay that demonstrates the utility of metabolomics for detection of lung cancer.

## 2. Patients and Methods

### 2.1. Study Population

**Figure 1:**
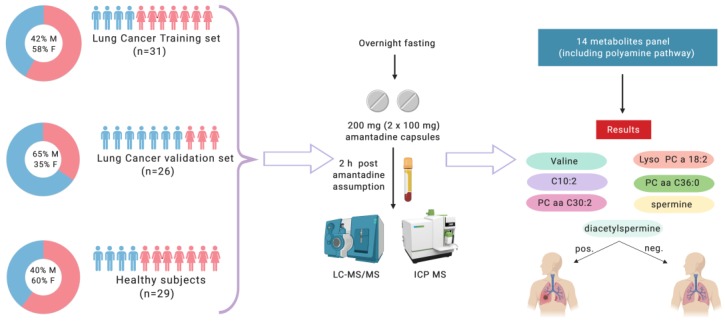
Study design. Credit: created with BioRender.

This study was undertaken to explore the use of amantadine as a means of measuring increased SSAT-1 activity. Patients with newly diagnosed and untreated cancer were recruited into the study. Lung cancer patients were recruited from the National Institute of Cancer Research & Hospital, Department of Medical Oncology, Mohakhali, Dhaka, Bangladesh. Healthy controls (*n* = 29) were recruited from within same geographical location. The subjects used for this study were derived from a lung cancer patient cohort (*n* = 80). All participants provided their approval with a signed informed consent for participation. Volunteers aged between 25 and 75 (median age: 52) years were included in the study (Figure 1). Exclusion criteria were declared as follows: alcohol consumption within 5 days of amantadine ingestion, previous adverse reaction to amantadine, currently pregnant or lactating, and liver or kidney disease. On the day of the study, blood samples were collected from overnight-fasted participants, prior to ingesting amantadine, and then requested to orally ingest 200 mg (2 × 100 mg) amantadine capsules (Mylan-Amantadine, amantadine hydrochloride, USP). Blood was collected 2 hours after amantadine administration. Following blood collection by venipuncture using sodium-oxalate coated vacutainer tubes, plasma was isolated by centrifugation (1000× *g* for 15 min) and plasma aliquoted (500 μL aliquots) and stored at −80 °C until analysis. 

The study was approved by the Institutional Review Board of the Ministry of Health & Family Welfare, the People’s Republic of Bangladesh (No. 115-15882). Clinical studies were completed under GCP and GLP conditions in accordance with local standards as well as the standards established by the Canadian Tri-Council Policies. 

### 2.2. Sample Analysis

In the present study, plasma samples 2 hours post-ingestion of 200 mg of amantadine were analyzed. A high-throughput DI/LC-MS/MS based targeted quantitative assay for plasma samples has been developed and applied to measure a total of 15 metabolites (The Metabolomics Innovation Centre, Edmonton, AB, Canada), i.e., valine, putrescine, MTA (5′-Methylthioadenosine), Arginine, Ornithine, Spermidine, spermine, di-acetyl spermine, methionine, SAMe, N-acetyl Amantadine, Decadienylcarnitine (C10:2), PC aa C32:2, PC ae C36:0, lysoPC a C18:2 in plasma samples (Supplementary Materials, Table S3). The samples were analyzed using a kit- Optima™ LC/MS grade formic acid and HPLC grade water were purchased from Fisher Scientific (Ottawa, ON, Canada). L-valine, putrescine, arginine, ornithine, spermidine, spermine, methylthioadenosine, methionine, ammonium acetate, phenylisothiocyanate (PITC), HPLC grade pyridine, HPLC grade ethanol and HPLC grade acetonitrile (ACN) were purchased from Sigma-Aldrich (Oakville, ON, Canada). 1,2-dipalmitoleoyl-sn-glycero-3-phosphocholine, 1,2-distearoyl-sn-glycero-3-phosphocholine, 1-(9Z,12Z-octadecadienoyl)-sn-glycero-3-phosphocholine were purchased from Avanti Polar Lipids, Inc. (Alabaster, AL, USA). N 1, N 12 -diacetylspermine (hydrochloride) was purchased from Cayman Chemical (Ann Arbor, Michigan, USA). Decadienylcarnitine was purchased from Medical Isotopes, Inc. (Pelham, NH, USA). Tableisotope-labelled standards, including d 8 -L-valine, 13 C 4 -1,4- butanediamine, 13 C 6 -arginine, and 5,5-d 2 -L-ornithine were purchased from Cambridge Isotope Laboratories, Inc. (Tewksbury, MA, USA). d 8 -spemidine, d 8 -spermine, and d 3 -decanoyl-L-carnitine were purchased from IsoSciences (Ambler, PA, USA). 15 N 5 -adenosine was purchased from Medical Isotopes, Inc. (Pelham, NH, USA). 1-linoleoyl-2-hydroxy-sn-glycero-3-phosphocholine-N, N, N-trimethyl-d 9 was purchased from Avanti Polar Lipids, Inc. (Alabaster, AL, USA). Multiscreen “solvinert” filter plates (hydrophobic, PTFE, 0.45 μm, clear, non-sterile) and Nunc™ 96 Deep Well™ plates were purchased from Sigma-Aldrich (Oakville, ON, Canada) based assay (96-well plate format).

We have applied a targeted quantitative metabolomics approach to analyze the samples using a combination of direct injection (DI) mass spectrometry with a reverse-phase LC-MS/MS Kit. This kit in combination with an ABI 4000 Q-Trap (Applied Biosystems/MDS Sciex) mass spectrometer were used for the targeted identification and quantification of metabolites. The method used combines the derivatization and extraction of analytes, and the selective mass-spectrometric detection using multiple reaction monitoring (MRM) pairs. Isotope-labeled internal standards and other internal standards are used for metabolite quantification. The kit contains a 96 deep-well plate with a filter plate attached with sealing tape, and reagents and solvents used to prepare the plate assay. First 14 wells in the Kit were used for one blank, three zero samples, seven standards and three quality control samples provided with each Kit. Briefly, samples were thawed on ice and were vortexed and centrifuged at 13,000× *g*. 10 µL of each sample was loaded onto the center of the filter on the upper 96-well kit plate and dried in a stream of nitrogen. Subsequently, phenyl-isothiocyanate was added for derivatization. After incubation, the filter spots were dried again using an evaporator. Extraction of the metabolites was then achieved by adding 300 µL of extraction solvent. The extracts were obtained by centrifugation into the lower 96-deep well plate, followed by a dilution step with kit MS running solvent. Mass spectrometric analysis was performed on an API4000 Qtrap^®^ tandem mass spectrometry instrument (Applied Biosystems/MDS Analytical Technologies, Foster City, Canada) equipped with a solvent delivery system. The samples were delivered to the mass spectrometer by a LC method followed by a direct injection (DI) method. Data analysis was done using Analyst 1.6.2.

### 2.3. Data Analysis

Raw metabolomics data were pre-processed, (1) the metabolites with more than 20% of missing values in all the groups were removed; (2) when missing values were less than 20%, they were imputed by half of minimum value for that specific metabolite. Two metabolites, methylthioadenosine (MTA) and S-adenosyl-L-methionine (SAMe), were removed from the analysis since they did not meet the quality control test. T-Test [33] has been applied to examine the metabolites varying among normal and cancerous patients and false discovery rate (fdr) [34] used for dealing with multiple testing error. Pearson’s correlation coefficient (r) was used to investigate and measure the strength between the different metabolites in the training and validation datasets. The prediction ability of lung cancer was measured by each metabolite independently (univariate approach) and in combination (multivariate approach) using generalized linear regression model. Performance of the model was measured by area under the curve (AUC, ROC) [35]. The robustness of outcome is evaluated using 10-fold cross validation. In order to examine if further improvements could be achieved by combining more variable(s) to the primary model (with just top variable), the following was undertaken: (1) All the variables were ranked according to their AUC value (high to low). (2) One by one, each variable added to the high ranked variable and improvement of AUC was monitored. Different permutations and combinations were applied to find the best predictor with highest prediction ability. The entire analysis was performed using R/Bio-conductor. (https://www.r-project.org/).

## 3. Results 

The participant characteristics that include details about number of samples and clinical factors such as age and cancer subtypes are provided in Supplementary Materials, Table S1. Supplementary Materials, Table S1A is for first lung cancer cohort group A (training dataset), while Supplementary Materials, Table S1B is the second cohort group B (validation dataset) which included mostly advanced stage (3+) lung cancers. Both cohorts A and B were from the *n* = 80 lung cancer samples. The present analysis was based on 57 of 80 lung cancer patients. For the baseline, a cohort of healthy volunteers was chosen from recruited patients of *n* = 29. Summary of both cancer and healthy cohorts is detailed in Supplementary Materials, Table S1C. A statistical summary of targeted and measured metabolites along with number of samples in both training and validation cohort datasets is provided in Supplementary Materials, Table S2. Targeted metabolites consist of polyamine and other endogenous metabolites comprised of amino acids, biogenic amines, acylcarnitines and glycerophospholipids as labelled in Supplementary Materials, Table S2.

### 3.1. Cluster Analysis and Correlation Matrix

**Figure 2:**
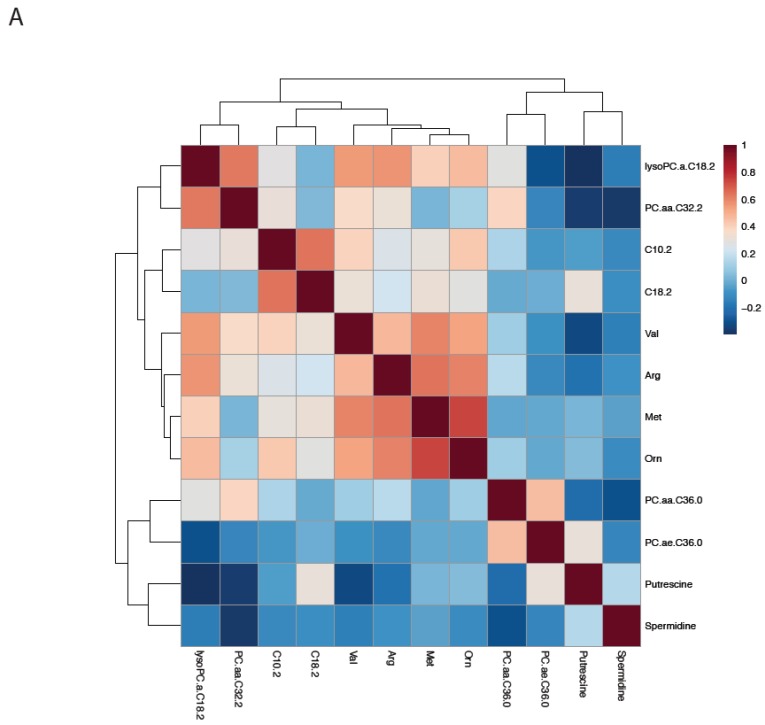

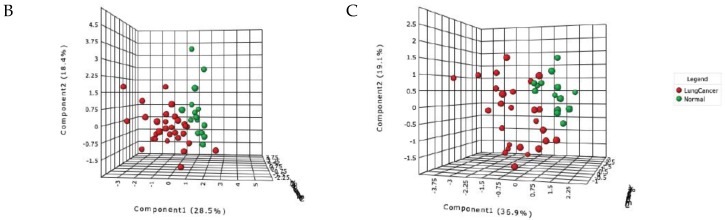
Cluster Analysis. Hierarchical cluster analysis and heat map of correlation between the metabolites using training dataset (**A**); Partial Least-Squares Discriminant Analysis: training dataset (**B**); and test dataset (**C**).

Hierarchical cluster analysis and heatmap of metabolite-metabolite correlation matrix was conducted and are shown in Figure 2A on training dataset. A popular machine learning tool Partial Least-Squares Discriminant Analysis (PLS-DA) was used as a classifier to measure the differentiation between normal and lung cancer patients. PLS-DA is a chemometrics technique that is used to optimize separation between different groups of samples. There is evidence of good separation as depicted in Figure 2B,C for both the training and validation cohort vs. the healthy group.

### 3.2. Univariate Results 

Each metabolite was tested independently to establish the prediction ability of outcome (lung cancer in this case) and then evaluated using training and validation datasets, respectively. Univariate summary between the datasets is presented in Supplementary Materials, Table S3A. Valine and lysoPhosphatidylcholine acyl C18:2 (lyso PC a C18.2) were the most significant metabolites with false discovery rate (fdr) < 0.01 for both the training and validation datasets when t-test analysis were performed. However, Decadienyl-L-carnitine (C10:2), Phosphatidylcholine diacyl C 32:2 (PC aa C32:2), Phosphatidylcholine diacyl C 36:0 (PC aa C36:0) and putrescine were significant in training data. Spermine and Diacetylspermine, which were detected only in validation data and showed significance at fdr < 0.05. A summary of the t tests for both the training and validation datasets is shown in Supplementary Materials, Table S3B.

### 3.3. Multivariate Results

**Figure 3:**
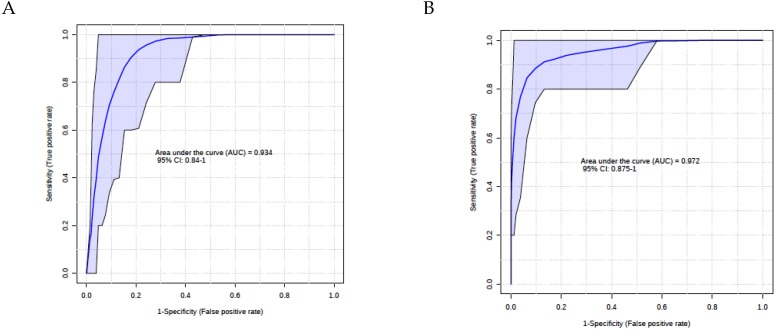
Linear regression multivariate modeling with multiple combinations of metabolites 5 metabolites included valine, putrescine, PC.ae.C36.0, PC.aa.C32.2 and C10.2 (**A**) and 3 key metabolites Valine, Spermine and Ornithine (**B**).

We tested multiple combinations of metabolites using linear regression multivariate modeling techniques to find the best predictor of lung cancer. Summary of the key metabolites is shown in Supplementary Materials, Table S3C (training) and Supplementary Materials, S3D (validation). Using training data maximum AUC ROC achieved was 0.93 (0.84–1.0) with 5 metabolites which included valine, putrescine, PC.ae.C36.0, PC.aa.C32.2 and C10.2, as shown in Figure 3A. In the validation data, maximum AUCROC achieved was 0.97 (0.86–1) with three key metabolites valine, spermine and ornithine (Figure 3B). Based on these results, we found valine as the common predictor of lung cancer in both univariate and multivariate setting. 

### 3.4. Box Plots 

**Figure 4:**
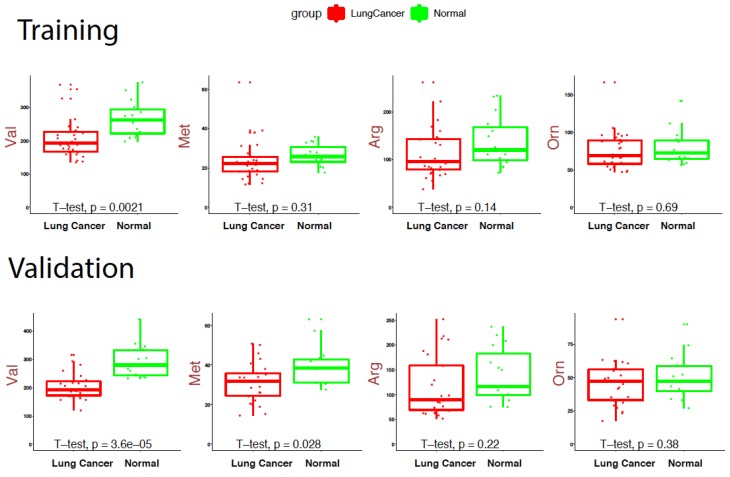
Box plots under training data and validation datasets. X axis represents disease status; Y axis represents metabolite concentrations of corresponding metabolite. Val: Valine; Met: Methionine; Arg: Arginine, Org: Ornithine. Supplementary Materials Figure S1 provides the full range of the Box Plots.

Box plots were constructed as a standardized approach to display the distribution concentrations of the different metabolites between lung cancer and healthy cohorts. In Figure 4, the Box Plots demonstrates the different metabolite concentration distributions for both training and validation data for a few selected metabolites.

## 4. Discussion

The purpose of this study was to evaluate whether combining a plasma panel of metabolites using a developed customized assay can further enhance and complement the SSAT-1 amantadine assay performance for detection of lung cancers. Patients that have tested with high levels of acetylamantadine can be further tested using this panel to better specify or rule in the type of cancer.

We utilized our finding to (1) identify a set of metabolites using a customized assay that may discriminate lung cancer from control following post-ingestion of amantadine (T2) and (2) understand the impact the new metabolites could have on improving the specificity of SSAT-1 amantadine assay. Analysis of the nature of the metabolites used for the discrimination of lung cancer from controls, as detailed earlier, reveals a panel of metabolites including valine, lysoPhosphatidylcholine acyl C18:2 (Lyso PC a18:2), decadienyl-L-carnitine (C10:2) phosphatidylcholine, acyl-alkyl C36:0 (PC aa C36:0), phosphatidylcholine diacyl C30:2 (PC aa C30:2), spermine, and diacetylspermine that can serve to discriminate between them. Changes in the concentrations of these metabolites are not surprising because lysophosphatidylcholines are membrane lipids known to be upregulated in lung cancer patients [36]. In addition, higher concentrations of amino acids are detected during lung tumor development [37,38]. For example, the high level of valine, leucine, and isoleucine found in lung tumors are required for energy production through the Krebs cycle [39]. Surprisingly, the metabolite diacetylspermine used in our study (even though only detected in the validation cohort) to discriminate patients from controls was found in plasma of patients as an excellent predictor of non-small cell lung cancer [40].

SSAT1, a key enzyme in the polyamine pathway (Figure 1), has been shown to be upregulated in different types of cancers [26,30,41,42,43]. In recently published studies, acetyltransferase activity of the enzyme demonstrated that it may be useful as a diagnostic test for lung cancer by monitoring the conversion of the drug amantadine to its acetylated form [31].

The results obtained from this study are generalizable/projectable, and are based mostly on lung cancer patients in stages 3+. The study has some limitations. Complete data related to smoking habits was not adequately collected. Confounding factors such as diet, exposure to natural arsenic in water, and overall pollution could be contributors to lung disease or cancer. Bangladesh’s contaminated well water is considered one of the largest public health crises in the world. An estimated 40 million people, approximately 25% of the population, are exposed to drinking water contaminated with arsenic (≥ 10 μg/L, the maximum levels allowed). The result is that trace arsenic exposure in Bangladesh appears to have led to dramatic increases in cancers ranging from skin to liver to lung, in cardiovascular disease, and in developmental and cognitive problems for children. However, the impact of environmental factors such as arsenic exposure in cancer development is complex, due to the long latency time before cancer development [44,45]. 

A separate study in a North American cohort (as well as other geographical regions) is warranted due to potential genetic and environmental influences. The authors are cognizant of these aspects and are accordingly in the process of completing a discovery, and a validation retrospective study based on a North American population using key learning from the present study has been planned by Rolfo et al. in collaboration with the University of Maryland. A larger retrospective study with emphasis on early detection, combining critical clinical parameters and pathology assessment reports may yield new insights and approach in early lung cancer diagnosis. In addition, understanding the metabolic pathways associated with these newly identified putative biomarkers and their role in lung cancer is warranted. 

## 5. Conclusions

The present study provides evidence that the customized assay, which was comprised of some metabolites corresponding to the polyamine pathway and other metabolites, is highly applicable and feasible. The collective panel amplifies the signal and increases the tissue specificity of the SSAT-1 amantadine assay, which may serve as a promising lung cancer diagnostic tool. The results show a clear need for a large-cohort study to confirm the findings for real world application.

**Clinical** **Practice Points**

- There is an urgent need to identify reliable, sensitive and economical diagnostic test for lung cancer, which is typically detected late and non-symptomatic where treatment options are limited and tend to be aggressive.
- Metabolomics is moving to clinical bedside with increased number of approved tests now being offered for diagnosis, prognosis and surveillance.
- We report in this paper a robust panel of 14 metabolites associated in the SSAT-1/polyamine pathway along with other endogenous metabolites comprised of amino acids, biogenic amines, acylcarnitines and glycerophospholipids amines that correctly discriminated between lung cancer patients from healthy controls using an established and customized assay.
- Detection and measurement of these specific metabolites can be employed to distinguish between healthy participants and patients with a diagnosis of lung cancer using existing LCMS equipment and infrastructure.
- This test can complement the SSAT1 Amantadine assay to further increase tissue specificity.

## Supplementary Materials

The following are available online at https://www.mdpi.com/2072-6694/11/8/1069/s1, Figure S1: The full range of the Box Plots, Table S1A: First lung cancer cohort group A (training dataset), Table S1B: First lung cancer cohort group B (validation dataset), Table S1C: Summary table of normal vs Lung cancer, Table S2: Statistical summary of samples and metabolite measures, Table S3A: Univariate Summary Measure of each Metabolite, Table S3B: T-test for key Metabolites using training data (A) and validation data, Table S3C: Training data, generalized linear regression multivariate model statistics-key metabolites, Table S3D: Validation data, generalized linear regression multivariate model statistics-key metabolites.

## References

[1] PDQ Adult Treatment Editorial Board (2002). Non-small cell lung cancer treatment (PDQ®): Health professional version. PDQ Cancer Information Summaries.

[2] Jemal A., Ward E.M., Johnson C.J., Cronin K.A., Ma J., Ryerson A.B., Mariotto A., Lake A.J., Wilson R., Sherman R.L. (2017). Annual Report to the Nation on the Status of Cancer, 1975–2014, Featuring Survival. J. Natl. Cancer Inst..

[3] Corrales L., Nogueira A., Passiglia F., Listi A., Caglevic C., Giallombardo M., Raez L., Santos E., Rolfo C. (2017). Second-Line Treatment of Non-Small Cell Lung Cancer: Clinical, Pathological, and Molecular Aspects of Nintedanib. Front. Med..

[4] Horeweg N., Scholten E.T., de Jong P.A., van der Aalst C.M., Weenink C., Lammers J.W., Nackaerts K., Vliegenthart R., ten Haaf K., Yousaf-Khan U.A. (2014). Detection of lung cancer through low-dose CT screening (NELSON): A prespecified analysis of screening test performance and interval cancers. Lancet Oncol..

[5] Gartman E.J., Jankowich M.D., Baptiste J., Nici L. Providence VA lung cancer screening program: Performance: Comparison of Local False Positive and Invasive Procedure Rates to Published Trial Data, A98. Clinical Strategies to Improve Lung Cancer Early Detection: Who is at Risk Here. American Journal of Respiratory and Critical Care Medicine, Proceedings of the American Thoracic Society International Conference Abstracts, San-Diego, CA, USA, 18–23 May 2018.

[6] Seijo L.M., Peled N., Ajona D., Boeri M., Field J.K., Sozzi G., Pio R., Zulueta J.J., Spira A., Massion P.P. (2019). Biomarkers in Lung Cancer Screening: Achievements, Promises, and Challenges. J. Thorac. Oncol..

[7] Mamdani H., Ahmed S., Armstrong S., Mok T., Jalal S.I. (2017). Blood-based tumor biomarkers in lung cancer for detection and treatment. Transl. Lung Cancer Res..

[8] Leygo C., Williams M., Jin H.C., Chan M.W.Y., Chu W.K., Grusch M., Cheng Y.Y. (2017). DNA Methylation as a Noninvasive Epigenetic Biomarker for the Detection of Cancer. Dise. Markers.

[9] Wishart S.D., Mandal R., Stanislaus A., Ramirez-Gaona M. (2016). Cancer Metabolomics and the Human Metabolome Database. Metabolites.

[10] Jordan K.W., Adkins C.B., Su L., Halpern E.F., Mark E.J., Christiani D.C., Cheng L.L. (2010). Comparison of squamous cell carcinoma and adenocarcinoma of the lung by metabolomic analysis of tissue–serum pairs. Lung Cancer.

[11] Hori S., Nishiumi S., Kobayashi K., Shinohara M., Hatakeyama Y., Kotani Y., Hatano N., Maniwa Y., Nishio W., Bamba T. (2011). A metabolomic approach to lung cancer. Lung Cancer.

[12] Mathé E.A., Patterson A.D., Haznadar M., Manna S.K., Krausz K.W., Bowman E.D., Shields P.G., Idle J.R., Smith P.B., Anami K. (2014). Noninvasive Urinary Metabolomic Profiling Identifies Diagnostic and Prognostic Markers in Lung Cancer. Cancer Res..

[13] Miyamoto S., Taylor L.S., Barupal K.D., Taguchi A., Wohlgemuth G., Wikoff R.W., Yoneda Y.K., Gandara R.D., Hanash M.S., Kim K. (2015). Systemic Metabolomic Changes in Blood Samples of Lung Cancer Patients Identified by Gas Chromatography Time-of-Flight Mass Spectrometry. Metabolites.

[14] Yokota H., Guo J., Matoba M., Higashi K., Tonami H., Nagao Y. (2007). Lactate, choline, and creatine levels measured by vitro 1H-MRS as prognostic parameters in patients with non-small-cell lung cancer. J. Magn. Reson. Imaging.

[15] Blackburn E.H. (2011). Cancer Interception. Cancer Prev. Res. (Phila).

[16] Tang Y., Li Z., Lazar L., Fang Z., Tang C., Zhao J. (2019). Metabolomics workflow for lung cancer: Discovery of biomarkers. Clin. Chim. Acta.

[17] Maes E., Mertens I., Valkenborg D., Pauwels P., Rolfo C., Baggerman G. (2015). Proteomics in cancer research: Are we ready for clinical practice?. Crit. Rev. Oncol. Hematol..

[18] Pegg A.E. (2016). Functions of Polyamines in Mammals. J. Biol. Chem..

[19] Babbar N., Hacker A., Huang Y., Casero R.A. (2006). Tumor Necrosis Factor α Induces Spermidine/Spermine N1-Acetyltransferase through Nuclear Factor κBin Non-small Cell Lung Cancer Cells. J. Biol. Chem..

[20] Gabrielson E., Tully E., Hacker A. (2004). Induction of spermidine/spermine N^1^-acetyltransferase in breast cancer tissues treated with the polyamine analogue N^1^, N^11^-diethylnorspermine. Cancer Chemother Pharmacol..

[21] Huang W., Eickhoff J.C., Mehraein-Ghomi F., Church D.R., Wilding G., Basu H.S. (2015). Expression of spermidine/spermine N1-acetyl transferase (SSAT) in human prostate tissues is related to prostate cancer progression and metastasis. Prostate.

[22] Pegg A.E. (2008). Spermidine/spermine-N1-acetyltransferase: A key metabolic regulator. Am. J. Phy. Endocrinol. Metab..

[23] Kingsnorth A.N., Wallace H.M. (1985). Elevation of monoacetylated polyamines in human breast cancers. Eu. J. Cancer Clin. Oncol..

[24] Pine M., Huben R., Pegg A. (1989). Production of N1-acetyl spermidine by renal cell tumors. J. Urol..

[25] Sessa A., Perin A. (1991). Increased synthesis of N1-acetylspermidine in hepatic preneoplastic nodules and hepatomas. Cancer Lett..

[26] YeA C., Bulovskaya L.N., Pavlova M.V., Krupkin R.G. (1978). Activity of N-acetyltransferase in patients with malignant lymphomas. Neoplasma.

[27] Bredel M., Bredel C., Juric D., Harsh G.R., Vogel H., Recht L.D., Sikic B.I. (2005). Functional network analysis reveals extended gliomagenesis pathway maps and three novel MYC-interacting genes in human gliomas. Cancer Res..

[28] Lee J., Kotliarova S., Kotliarov Y., Li A., Su Q., Donin N.M., Pastorino S., Purow B.W., Christopher N., Zhang W. (2006). Tumor stem cells derived from glioblastomas cultured in bFGF and EGF more closely mirror the phenotype and genotype of primary tumors than do serum-cultured cell lines. Cancer Cell.

[29] Sun L., Hui A.M., Su Q., Vortmeyer A., Kotliarov Y., Pastorino S., Passaniti A., Menon J., Walling J., Bailey R. (2006). Neuronal and glioma-derived stem cell factor induces angiogenesis within the brain. Cancer Cell.

[30] Bras A.P., Hoff H.R., Aoki F.Y., Sitar D.S. (1998). Amantadine acetylation may be effected by acetyltransferases other than NAT1 or NAT2. Can. J. Physiol. Pharmacol..

[31] Maksymiuk A.W., Sitar D.S., Ahmed R., Cheng B., Bach H., Bagchi R.A., Aroutiounova N., Tappia P.S., Ramjiawan B. (2018). Spermidine/spermine N1-acetyltransferase-1 as a diagnostic biomarker in human cancer. Future Sci. OA.

[32] Maksymiuk A.W., Tappia P.S., Sitar D.S., Akhtar P.S., Khatun N., Parveen R., Ahmed R., Ahmed R.B., Cheng B., Huang G. (2019). Use of amantadine as substrate for SSAT-1 activity as a reliable clinical diagnostic assay for breast and lung cancer. Future Sci. OA.

[33] Mankiewicz R. (2004). The Story of Mathematics.

[34] Yoav B., Hochberg Y. (1995). Controlling the false discovery rate: A practical and powerful approach to multiple testing. J. R. Stat. Soc. Ser. B.

[35] Hanley J.A. (1989). Receiver operating characteristic (ROC) methodology: The state of the art. Crit. Rev. Diagn. Imaging.

[36] Li Y., Song X., Zhao X., Zou L., Xu G. (2014). Serum metabolic profiling study of lung cancer using ultra high performance liquid chromatography/quadrupole time-of-flight mass spectrometry. J. Chromatogr. B Anal. Technol. Biomed. Life Sci..

[37] Fan T.W.M., Lane A.N., Higashi R.M., Farag M.A., Gao H., Bousamra M., Miller D.M. (2009). Altered regulation of metabolic pathways in human lung cancer discerned by (13) C stable isotope-resolved metabolomics (SIRM). Mol. Cancer.

[38] Kami K., Fujimori T., Sato H., Sato M., Yamamoto H., Ohashi Y., Sugiyama N., Ishihama Y., Onozuka H., Ochiai A. (2013). Metabolomic profiling of lung and prostate tumor tissues by capillary electrophoresis time-of-flight mass spectrometry. Metabolomics.

[39] Hirayama A., Kami K., Sugimoto M., Sugawara M., Toki N., Onozuka H., Kinoshita T., Saito N., Ochiai A., Tomita M. (2009). Quantitative metabolome profiling of colon and stomach cancer microenvironment by capillary electrophoresis time-of-flight mass spectrometry. Cancer Res..

[40] Wikoff W.R., Hanash S., DeFelice B., Miyamoto S., Barnett M., Zhao Y., Goodman G., Feng Z., Gandara D., Fiehn O. (2015). Diacetylspermine Is a Novel Prediagnostic Serum Biomarker for Non-Small-Cell Lung Cancer and Has Additive Performance With Pro-Surfactant Protein B. J. Clin. Oncol..

[41] Bras A.P., Janne J., Porter C.W., Sitar D.S. (2001). Spermidine/spermine n(1)-acetyltransferase catalyzes amantadine acetylation. Drug Metab. Dispos..

[42] Battaglia V., DeStefano Shields C., Murray-Stewart T., Casero R.A.J. (2014). Polyamine catabolism in carcinogenesis: Potential targets for chemotherapy and chemoprevention. Amino Acids.

[43] Takenoshita S., Matsuzaki S., Nakano G., Kimura H., Hoshi H., Shoda H., Nakamura T. (1984). Selective elevation of the N1-acetylspermidine level in human colorectal adenocarcinomas. Cancer Res..

[44] https://undark.org/article/bangladesh-arsenic-poisoning-drinking-water/.

[45] Soza-Ried C., Bustamante E., Caglevic C., Rolfo C., Sirera R., Marsiglia H. (2019). Oncogenic role of arsenic exposure in lung cancer: A forgotten risk factor. Crit. Rev. Oncol. Hematol..

