# Supplementary material for: Liquid Biopsy in Lung Cancer Screening: The Contribution of Metabolomics. Results of A Pilot Study
Source: Cancers (Basel). 2019 Jul 29;11(8):1069. doi: 10.3390/cancers11081069 (PMC6721278; doi:10.3390/cancers11081069)
Supplement: Supplementary file 1 [file cancers-11-01069-s001.pdf]

Supplementary materials

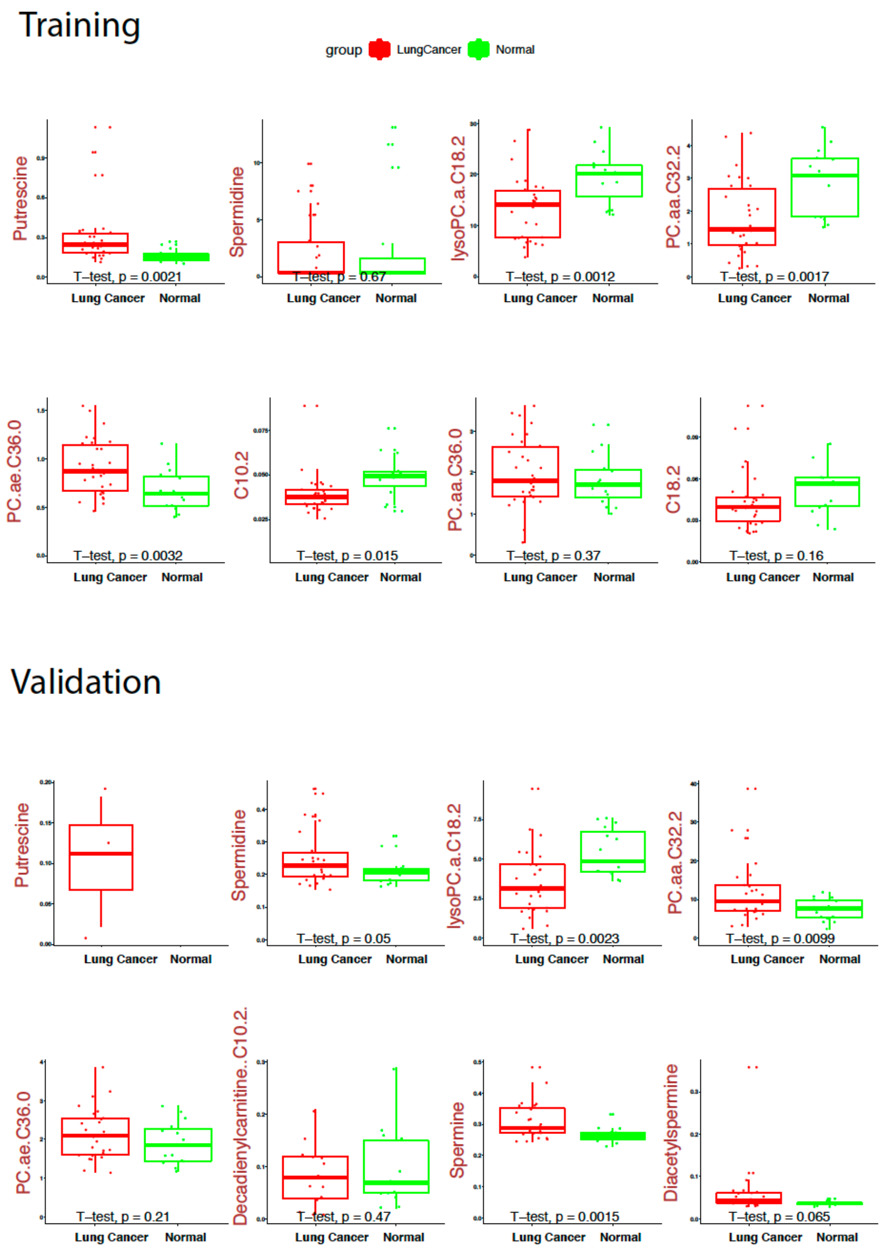

Figure 1. The full range of the Box Plots.

Table S1

Table 1A: Training cohort

Lung cancer subtypes						Age (yr)
	Adenocarcinoma (n)	Squamous (n)	Non Squamous (n)	Small cell (n)	Metastasis (n)	Mean±SD
Female (n=18)	13	5	-	-	-	47±2
Male (n=13)	9	3	-	1	-	56±2
Total (n= 31)	22	8	-	1	-	52±2

** Majority of patient at advanced stage 3+

Table 1B: Validation cohort

Lung cancer subtypes						Age (yr)
	Adenocarcinoma (n)	Squamous (n)	Non Squamous (n)	Small cell (n)	Metastasis (n)	Mean±SD
Female (n=9)	2	2	1	2	2	54±3
Male (n=17)	4	5	5	3	-	56±3
Total (n=26)	6	7	6	5	2	56±2

** Majority of patient at advanced stage 3+

Table 1C: Summary table of normal vs Lung cancer

	Tranining			Validation		
	Lung Cancer (n)	Normal (n)	Pvalue	Lung Cancer (n)	Normal (n)	Pvalue
Samples	31	15		26	15	
Age:	Mean±SD	Mean±SD		Mean±SD	Mean±SD	
Mean (Range) (yr)	50.7 (27 - 75)	43.7 (27 -56)	0.06	55.7 (27 - 70)	43.7 (27 -56)	0.000213
Sex:						
Male	13	6	0.75	17	6	0.12
Female	18	9		9	9	

Table S2

Table 2: Statistical summary of samples and metabolite measures

**Targeted metabolites**	Metabolites Classification	Patient Classification	Cohort	Number of samples	Number of missing samples	min value of metabolite	max value of metabolite	Median	Mean
Valine	**Amino Acids and Biogenic Amines**	Normal	Training	15	0	198	376	263	267.0667
Putrescine	**Polyamine Metabolites**	Normal	Training	15	0	0.104	0.269	0.154	0.1637
Methionine	**Polyamine Metabolites**	Normal	Training	15	0	17.7	35.9	25.8	26.5133
Arginine	**Polyamine Metabolites**	Normal	Training	15	0	72.4	234	120	137.3933
Ornithine	**Polyamine Metabolites**	Normal	Training	15	0	56.6	142	72.5	79.14
Spermidine	**Polyamine Metabolites**	Normal	Training	15	0	0.256	13.1	0.335	2.7091
C10.2	**Acylcarnitines**	Normal	Training	15	0	0.0297	0.076	0.0493	0.0489
C18.2	**Acylcarnitines**	Normal	Training	15	0	0.0238	0.0853	0.0567	0.0522
lysoPC.a.C18.2	**Glycerophospholipids**	Normal	Training	15	0	12.1476	29.259	20.1631	19.521
PC.aa.C32.2	**Glycerophospholipids**	Normal	Training	15	0	1.5099	4.5478	3.0779	2.9107
PC.aa.C36.0	**Glycerophospholipids**	Normal	Training	15	0	1.008	3.1557	1.7134	1.803
PC.ae.C36.0	**Glycerophospholipids**	Normal	Training	15	0	0.4003	1.157	0.6427	0.6749
Valine	**Amino Acids and Biogenic Amines**	Lung Cancer	Training	31	0	135	369	193	208
Putrescine	**Polyamine Metabolites**	Lung Cancer	Training	31	0	0.115	1.13	0.2475	0.3099
Methionine	**Polyamine Metabolites**	Lung Cancer	Training	31	0	11.6	63.6	22.25	24.0533
Arginine	**Polyamine Metabolites**	Lung Cancer	Training	31	0	37.7	262	95.6	112.2867
Ornithine	**Polyamine Metabolites**	Lung Cancer	Training	31	0	47.1	167	69.35	76.13
Spermidine	**Polyamine Metabolites**	Lung Cancer	Training	31	0	0.333	9.9	0.335	2.14
C10.2	**Acylcarnitines**	Lung Cancer	Training	31	0	0.0255	0.0889	0.0376	0.039
C18.2	**Acylcarnitines**	Lung Cancer	Training	31	0	0.0208	0.1127	0.0398	0.0438
lysoPC.a.C18.2	**Glycerophospholipids**	Lung Cancer	Training	31	0	3.815	28.7725	14.1162	13.3528
PC.aa.C32.2	**Glycerophospholipids**	Lung Cancer	Training	31	0	0.2588	4.3734	1.4442	1.78149
PC.aa.C36.0	**Glycerophospholipids**	Lung Cancer	Training	31	0	0.3143	3.6154	1.8023	2.0029
PC.ae.C36.0	**Glycerophospholipids**	Lung Cancer	Training	31	0	0.4613	1.5473	0.8711	0.9148
Valine	**Amino Acids and Biogenic Amines**	Normal	Validation	15	0	233	442	280	294.5333
Arginine	**Polyamine Metabolites**	Normal	Validation	15	0	75.1	237	116	139.88
Ornithine	**Polyamine Metabolites**	Normal	Validation	15	0	27	90	47.3	50.7533
Methionine	**Polyamine Metabolites**	Normal	Validation	15	0	27.5	63.1	38.4	39.02
Spermidine	**Polyamine Metabolites**	Normal	Validation	15	0	0.164	0.319	0.208	0.2118
Spermine	**Polyamine Metabolites**	Normal	Validation	15	0	0.23	0.332	0.263	0.2655
Diacetylspermine	**Acylcarnitines**	Normal	Validation	15	0	0.0287	0.0473	0.0364	0.0369
Decadienylcarnitine (C10.2)	**Acylcarnitines**	Normal	Validation	13	2	0.02	0.29	0.07	0.1008
PC.aa.C32.2	**Glycerophospholipids**	Normal	Validation	15	0	2.37	11.89	7.69	7.3293
PC.ae.C36.0	**Glycerophospholipids**	Normal	Validation	15	0	1.17	2.86	1.85	1.9033
lysoPC.a.C18.2	**Glycerophospholipids**	Normal	Validation	15	0	3.63	7.59	4.87	5.4073
MTA	**Polyamine Metabolites**	Lung Cancer	Validation	9	17	0.0041	0.267	0.0945	0.1067
Valine	**Amino Acids and Biogenic Amines**	Lung Cancer	Validation	26	0	120	316	192	204.6154
Arginine	**Polyamine Metabolites**	Lung Cancer	Validation	26	0	51.6	252	89.8	116.8347
Ornithine	**Polyamine Metabolites**	Lung Cancer	Validation	26	0	17.4	93.7	47.15	45.9423
Methionine	**Polyamine Metabolites**	Lung Cancer	Validation	26	0	14.5	50.8	31.8	31.4423
Putrescine	**Polyamine Metabolites**	Lung Cancer	Validation	3	23	0.0219	0.182	0.112	0.1053
Spermidine	**Polyamine Metabolites**	Lung Cancer	Validation	26	0	0.154	0.464	0.2285	0.2531
Spermine	**Polyamine Metabolites**	Lung Cancer	Validation	26	0	0.245	0.482	0.2885	0.3108
Diacetylspermine	**Acylcarnitines**	Lung Cancer	Validation	26	0	0.0297	0.358	0.0423	0.0611
Decadienylcarnitine (C10.2)	**Acylcarnitines**	Lung Cancer	Validation	15	11	0.01	0.21	0.08	0.0813
PC.aa.C32.2	**Glycerophospholipids**	Lung Cancer	Validation	26	0	3.08	38.67	9.48	12.1392
PC.ae.C36.0	**Glycerophospholipids**	Lung Cancer	Validation	26	0	1.14	3.86	2.095	2.1485
lysoPC.a.C18.2	**Glycerophospholipids**	Lung Cancer	Validation	26	0	0.6	9.44	3.13	3.5692

Table S3

Table 3A: Univariate Summary Measure of each Metabolite

	Training			Validation		
	AUC	P value	FC	AUC	P value	FC
Arginine	6.61E-01	1.30E-01	-3.25E-01	6.64E-01	1.33E-01	-3.26E-01
C10.2	7.71E-01	9.44E-03	-1.28E-01	NA	NA	NA
C18.2	6.73E-01	1.85E-01	-1.11E-01	NA	NA	NA
Diacetylspermi	NA	NA	NA	7.60E-01	2.59E-02	2.12E-01
lysoPC.a.C18.2	7.73E-01	1.86E-03	-6.60E-01	7.65E-01	3.16E-03	-8.15E-01
Methionine	6.67E-01	4.01E-01	-2.19E-01	6.85E-01	2.08E-02	-3.44E-01
Ornithine	5.46E-01	6.97E-01	-6.91E-02	5.69E-01	3.21E-01	-1.70E-01
PC.aa.C32.2	7.96E-01	2.12E-03	-9.63E-01	6.94E-01	2.75E-02	5.91E-01
PC.aa.C36.0	5.71E-01	4.13E-01	7.44E-02	NA	NA	NA
PC.ae.C36.0	7.51E-01	6.69E-03	4.28E-01	6.12E-01	2.53E-01	1.64E-01
Putrescine	8.33E-01	2.03E-02	6.64E-01	NA	NA	NA
Spermidine	5.89E-01	6.12E-01	2.46E-01	6.29E-01	1.11E-01	1.90E-01
Spermine	NA	NA	NA	7.67E-01	5.20E-03	1.95E-01
Valine	8.04E-01	2.19E-03	-3.83E-01	9.09E-01	2.58E-06	-5.33E-01

AUC – Area Under the Curve; FC – Fold Change, NA - Not available

Table 3B: t-tests for key Metabolites using training data (A) and validation data (B)

**Training Data A**				
**Metabolites**	**t-statistic**	**P value**	**-log10 (p)**	**FDR**
Putrescine	3.54E+00	9.88E-04	3.01E+00	6.44E-03
Valine	-3.51E+00	1.07E-03	2.97E+00	6.44E-03
lysoPC.a.C18.2	3.30E+00	1.97E-03	2.70E+00	7.89E-03
PC.aa.C32.2	-3.18E+00	2.72E-03	2.57E+00	8.15E-03
PC.ae.C36.0	3.02E+00	4.27E-03	2.37E+00	9.32E-03
C10.2	-2.99E+00	4.66E-03	2.33E+00	9.32E-03
**Validation Data B**				
**Metabolites**	**t-statistic**	**P value**	**-log10 (p)**	**FDR**
Valine	-5.50E+00	2.58E-06	5.59E+00	2.58E-05
lysoPC.a.C18.2	-3.15E+00	3.16E-03	2.50E+00	1.58E-02
Spermine	2.96E+00	5.20E-03	2.28E+00	1.73E-02
Methionine	-2.41E+00	2.08E-02	1.68E+00	4.59E-02
Diacetylspermine	2.32E+00	2.59E-02	1.59E+00	4.59E-02
PC aa. C32:2	2.29E+00	2.75E-02	1.56E+00	4.59E-02

-log10 (p), FDR False Discovery Rate; t-statistic,

Table 3C: Training data, generalized linear regression multivariate model statistics – key metabolites

	**Estimate**	**S.E.**	**P value**
(Intercept)	0.9438	0.2969	0.0029
Valine	0.0012	0.0011	0.2724
Putrescine	-0.6203	0.2991	0.0447
PC.aa.C32.2	0.1294	0.0563	0.027
PC.aa.C36.0	-0.2273	0.08	0.0071
C10.2	10.3848	5.2603	0.0555

Table 3D: Validation data, generalized linear regression multivariate model statistics – key metabolites

	**Estimate**	**S. E.**	**P Value**
(Intercept)	1.3407	0.4139	0.0025
Valine	0.0051	0.0009	1.84E-06
Spermine	-2.8954	1.0274	0.0077
Ornithine	-0.007	0.0036	0.0633

S.E., Standard Error
